# Metabolic Remodeling in Peritoneal Dialysis-Associated Peritoneal Fibrosis: Mechanisms, Candidate Metabolic Signatures, and Therapeutic Perspectives

**DOI:** 10.3390/metabo16100724

**Published:** 2026-09-28

**Authors:** Zhengjie Zhu, Mengyang Wang, Tao Zhang, Jie Li, Bo Yang, Hongtao Yang

**Affiliations:** 1 First Teaching Hospital of Tianjin University of Traditional Chinese Medicine, Tianjin 300381, China; zhuzhengjie1989@163.com (Z.Z.); zhangtao06653@163.com (T.Z.); lijie860303@163.com (J.L.); 2 National Clinical Research Center for Chinese Medicine, Tianjin 300381, China; 3 School of Public Health and Health Sciences, Tianjin University of Traditional Chinese Medicine, Tianjin 300193, China; mengyangwang@tjutcm.edu.cn

**Keywords:** peritoneal dialysis, peritoneal fibrosis, metabolic remodeling, metabolomics, lipidomics, glycolysis, mitochondrial dysfunction, ferroptosis, immunometabolism, candidate metabolic signatures

## Abstract

Peritoneal fibrosis is a major cause of progressive peritoneal membrane dysfunction and technique failure in patients receiving long-term peritoneal dialysis. Conventional models emphasize repeated chemical injury, sterile inflammation, angiogenesis, and transforming growth factor-β signaling, but they do not fully explain why apparently similar dialysis exposures produce different trajectories of membrane loss. Here, we develop a metabolism-centered framework in which glucose-rich dialysate, glucose degradation products, carbonyl stress, hypoxia, and inflammatory mediators may establish a maladaptive metabolic state during membrane remodeling. Available evidence supports an important and increasingly recognized metabolic dimension of PD-associated membrane injury, although direct human evidence for structural PF remains limited. Evidence from peritoneal dialysis models and patient effluent studies supports coordinated changes in glycolysis, mitochondrial oxidative metabolism, fatty-acid oxidation, lipid peroxidation, amino-acid anaplerosis, and immune-cell metabolism. These changes are associated with mesothelial-to-mesenchymal transition, macrophage plasticity, fibroblast activation, extracellular-vesicle signaling, and ferroptosis-related or inflammasome-associated injury. We summarize LC–MS-, GC–MS-, and NMR-based metabolomics, proteomics, and integrated multi-omics approaches for characterizing dialysate and plasma signatures, and we define a staged validation pathway from discovery to longitudinal clinical prediction. The study-level evidence tables are selected narrative evidence maps that separate human membrane-function or inflammatory signals from experimental structural fibrosis endpoints and report the model, exposure, quantitative result, and principal limitation for each cited study. Therapeutic opportunities include biocompatible dialysis-fluid design, glycolytic and mitochondrial modulation, restoration of redox and lipid homeostasis, ferroptosis control, immunometabolic interventions, and rational evaluation of bioactive compounds. The central translational hypothesis is that serial dialysate metabolomics may identify a reversible phase of membrane injury before fixed fibrosis; this hypothesis requires prospective validation against structural or clinically adjudicated peritoneal fibrosis outcomes.

## 1. Introduction

Peritoneal dialysis (PD) provides home-based kidney replacement therapy and can preserve treatment autonomy, residual kidney function, and vascular access options. Its long-term effectiveness depends on the integrity of the peritoneal membrane, which must repeatedly accommodate osmotic, chemical, mechanical, and immunological stress. Non-infectious complications, including progressive solute transport, ultrafiltration failure, encapsulating peritoneal sclerosis (EPS), and loss of membrane integrity, are clinically heterogeneous and often emerge after years of exposure rather than after a single acute insult [1,2,3]. Histological studies link these phenotypes to mesothelial denudation, submesothelial thickening, extracellular-matrix (ECM) accumulation, vascular remodeling, and chronic inflammatory infiltration. Contemporary reviews of PD fluids and membrane biology emphasize that solution chemistry and inflammatory tone jointly shape long-term membrane outcomes [4,5]. However, the upstream events that determine whether peritoneal injury resolves or becomes self-sustaining remain incompletely defined.

The conventional injury–inflammation–fibrosis model remains useful, but it is not metabolically neutral. Standard glucose-based solutions deliver a repeated carbon load; heat sterilization generates glucose degradation products (GDPs); acidic or hyperosmolar solutions perturb cell volume and redox balance; and systemic glucose absorption can worsen insulin resistance and dyslipidemia. These exposures alter the availability, routing, and redox state of metabolites in mesothelial cells, macrophages, fibroblasts, endothelial cells, and circulating immune cells. In experimental systems, metabolic stress can operate upstream of, and in parallel with, inflammatory signaling: altered substrate flux changes reactive oxygen species (ROS), lactate, lipid mediators, amino-acid anaplerosis, and damage-associated molecular patterns (DAMPs), which may then reinforce transforming growth factor (TGF)-β, nuclear factor-κB (NF-κB), signal transducer and activator of transcription 3 (STAT3), and inflammasome pathways [6,7,8]. Clinical and experimental syntheses likewise describe oxidative stress and sterile inflammation as coupled rather than independent processes in PD [9,10]. Direct longitudinal human evidence that metabolic remodeling temporally precedes structural PF is not yet available.

An earlier systematic review in *Metabolites* evaluated metabolomics technologies for characterizing peritoneal membrane profiles [11]. The present review has a different organizing question: whether pathway-level metabolic remodeling can connect dialysis-fluid exposure to multicellular fibrotic remodeling and, ultimately, to testable candidate metabolic signatures and interventions. A cognitive-aging cohort study suggests that pathway-level metabolomic patterns may complement individual analytes when summarizing complex biological states; this methodological consideration may inform candidate PD-signature design but does not constitute direct evidence for peritoneal fibrosis [12]. We, therefore, integrate metabolic flux and pathway perturbation, glycolysis–mitochondria–ferroptosis crosstalk, immunometabolism, extracellular-vesicle signaling, and study-level separation of human surrogate endpoints from experimental structural PF. We focus on five connected metabolic modules: (i) glucose metabolism and glycolytic remodeling, (ii) mitochondrial dysfunction and oxidative metabolism, (iii) lipid metabolism and ferroptosis, (iv) amino-acid and tricarboxylic-acid (TCA) cycle remodeling, and (v) immunometabolic plasticity. We then map these modules onto multicellular communication and discuss how metabolomics could support risk stratification and precision preservation of the peritoneal membrane after prospective validation.

## 2. Review Methodology

### 2.1. Information Sources and Search Architecture

We performed a structured narrative evidence-mapping search in PubMed/MEDLINE, Elsevier Embase, Web of Science Core Collection, and Google Scholar. Searches covered database inception through 26 August 2026; Google Scholar was used primarily for forward and backward citation chasing and terminology expansion. Two parallel search streams were used. Stream A combined PD with PF, membrane dysfunction, or EPS and metabolic mechanisms such as glycolysis, mitochondrial dysfunction, lipidomics, ferroptosis, amino-acid metabolism, or immunometabolism. Stream B combined PD, dialysate, or effluent with metabolomics, lipidomics, metabolites, proteomics, or extracellular vesicles without requiring the term “fibrosis”; this stream was designed to capture human studies of transport, peritonitis, and pharmacodynamic responses that could inform membrane biology without constituting PF biomarker studies. The compact database-adapted search blocks are provided in Appendix A.

Records were screened for relevance to PD-associated peritoneal injury, fibrosis, membrane transport, effluent markers, EPS, metabolic pathways, or analytical validation. Duplicate records were removed using DOI, PMID, title, and first-author matching, followed by title/abstract screening and full-text assessment when needed. Four authors participated in literature curation, and uncertain inclusion or evidence-tier decisions were resolved by author-team consensus; this narrative review did not use independent duplicate screening or a registered protocol. The human evidence table includes studies for which a concrete metabolite or multi-omic readout and a quantitative membrane, inflammatory, pharmacodynamic, or systemic PD endpoint could be extracted; it is a deliberately heterogeneous evidence map rather than a pooled diagnostic dataset. The final synthesis included 108 references spanning human PD studies, experimental PD models, analytical-method papers, and mechanistic studies from related renal-fibrosis fields. Because complete per-database hit counts and a prospectively logged screening flow were not retained, we do not present a PRISMA flow diagram or pooled effect estimate.

For the study-level evidence tables, we extracted one primary study per row and prioritized numerical fields in the following order: biological sample size, exposure concentration or intervention dose, treatment duration, effect estimate or absolute/relative change, uncertainty interval, and exact or thresholded *p* value. When a paper reported several assays, the in-vivo structural or peritoneal-function result was prioritized over molecular-marker changes. Values shown only graphically were not digitized, and no effect size was calculated from images; such fields are reported as “NR” rather than estimated. Sample sizes refer to biological replicates or participants when this distinction was stated in the primary study. Heterogeneous endpoints and units were not pooled.

### 2.2. Eligibility and Evidence Interpretation

Studies were prioritized when they met at least one of the following criteria: (1) direct investigation of PD-associated peritoneal injury, fibrosis, transport, effluent, or EPS; (2) measurement of metabolites, metabolic enzymes, metabolic flux, or omics signatures in PD patients, dialysate, peritoneal tissue, or relevant experimental models; (3) mechanistic experiments linking a metabolic node to mesothelial, immune, endothelial, or fibroblast behavior; or (4) validated kidney-fibrosis or immunometabolism evidence that could be explicitly labeled as mechanistic extrapolation. Within this eligible corpus, primary studies were selected for Table 1, Table 2, Table 3 and Table 4 according to quantitative extractability, mechanistic relevance, relevance of the measured endpoint, evidence strength or recency, and complementary coverage of the review’s major pathways. Table 1, Table 2, Table 3 and Table 4 are transparent, purposefully selected narrative evidence maps rather than exhaustive systematic-study inventories or pooled quantitative syntheses. Human observational studies were used primarily for biomarker feasibility and association, whereas animal and cell studies were used to discuss causality and therapeutic perturbation.

### 2.3. Evidence Hierarchy and Terminology

To prevent mechanistic plausibility from being interpreted as PD-specific causality, evidence was assigned to five mutually exclusive tiers: (I) human PD evidence with histological, validated structural imaging, or clinically adjudicated PF/EPS endpoints; (II) human PD evidence with functional or surrogate membrane endpoints, including transport, ultrafiltration, inflammation, peritonitis, or pharmacodynamic responses; (III) experimental PD or peritoneal-fibrosis models with pathway perturbation and a structural or functional peritoneal endpoint; (IV) mechanistic evidence extrapolated from other renal-fibrosis or kidney-injury models; and (V) proposed or hypothesis-generating mechanisms without direct PD validation. In this review, “validated structural imaging” means protocolized cross-sectional imaging (for example, CT or MRI, when a study has prespecified criteria for peritoneal thickening, calcification, encapsulation, or related structural change and independent image adjudication) that has been evaluated against a reference standard or clinically adjudicated PF/EPS outcome. No single imaging modality is assumed to be a universally validated substitute for histology. These tiers encode proximity and directness to PD-associated structural PF, not overall methodological quality: a small direct peritoneal study may occupy a higher tier than a larger, methodologically stronger but indirect study. Histological PF, structural or imaging fibrosis, EPS, ultrafiltration failure, PET transport phenotype, peritonitis, and pharmacodynamic metabolic responses are related but non-interchangeable outcomes. In this review, “PF specificity” is an evidentiary claim rather than a synonym for PF relevance: a candidate is called PF-specific only when its association or trajectory is tied to a Tier I structural PF/EPS outcome, persists after prespecified adjustment for residual kidney function, dialysis vintage, solution glucose strength, fill and dwell parameters, ultrafiltration, transport phenotype, peritonitis/antibiotic exposure, systemic inflammation and paired plasma concentration, and is independently replicated. Signals associated only with PET transport, ultrafiltration failure, peritonitis, inflammation, or a pharmacodynamic response are described by that endpoint and are not called PF-specific. No human effluent study in the selected map currently meets all of these criteria. The term “PF biomarker” is reserved for a marker validated against a Tier I outcome; all other human signals are described by their measured endpoint. Direct causal language is reserved for perturbation studies with appropriate rescue or target-engagement evidence. Evidence domains are displayed separately and are not summed into a quality score.

## 3. The PD Environment as a Metabolic Stressor

### 3.1. Glucose Load, GDPs, and Pseudohypoxia

Glucose is an effective osmotic agent but creates a repeated metabolic exposure. Glucose absorbed from dialysate contributes to systemic caloric load and can aggravate dysglycemia, hyperinsulinemia, and dyslipidemia; a meta-analysis has documented incident glucose disorders in PD populations [13,14]. At the peritoneal surface, high glucose increases glucose uptake and glycolytic flux in mesothelial and endothelial cells. The resulting rise in pyruvate and lactate is not simply a marker of cellular stress: lactate can act as a signaling metabolite, alter extracellular pH, stabilize hypoxia-inducible factor-1α (HIF-1α), and influence chromatin and immune-cell function.

Among the most direct mechanistic evidence for a metabolism-driven process is work showing that inhibition of hyperglycolysis in mesothelial cells prevents experimental peritoneal fibrosis [6]. Complementary studies link high-glucose exposure to STAT3/HIF-1α activation, mesothelial-to-mesenchymal transition (MMT), and increased expression of TGF-β, vascular endothelial growth factor, and ECM genes [8,15]. A recent analysis of endothelial cells similarly reported that hyperglycolysis is associated with endothelial injury and microvascular alterations in PD [16]. Together, these observations are consistent with the testable hypothesis that a glycolytic switch may occur early enough to couple glucose availability to hypoxia-like signaling and vascular remodeling; longitudinal human data are still lacking.

GDPs and advanced glycation end products (AGEs) add a second layer of metabolic stress. GDPs are generated during heat sterilization, and carbonyl species such as methylglyoxal can modify proteins, deplete glutathione, and activate AGE receptors. In a clinical and experimental framework, glucose-induced pseudohypoxia and AGE accumulation were proposed as complementary explanations for peritoneal membrane dysfunction [17]. Methylglyoxal-induced peritoneal injury is accompanied by hypermetabolism of glutathione, glutamate, and ornithine, indicating that amino-acid and redox pathways respond together rather than independently [18]. Studies of GDP-exposed endothelial and vascular smooth-muscle cells further link solution-derived carbonyls to impaired angiogenic signaling [19]. The practical implication is that reducing glucose concentration alone may not remove the carbonyl burden if GDP generation and buffer chemistry remain unfavorable.

### 3.2. Oxidative, Carbonyl, and Osmotic Stress

Mesothelial cells are exposed to high osmolality, low pH, lactate, GDPs, and intermittent mechanical stretch. These inputs increase mitochondrial and cytosolic ROS, oxidize membrane lipids and proteins, and consume reduced glutathione. Reviews of PD fluids consistently identify oxidative stress as a convergent pathway linking solution chemistry to mesothelial injury and membrane dysfunction [20,21,22]. The TXNIP/TRX axis and other thiol-redox systems provide measurable links between oxidative DNA damage and the PD state [23,24]. Broader PD and kidney-injury reviews place these observations within a redox–inflammation continuum and support measurement of glutathione, thioredoxin, and lipid-oxidation products as complementary readouts [10,25].

Oxidative stress is not synonymous with irreversible injury. Mitochondrial quality control, glutathione synthesis, NADPH regeneration, and autophagy can buffer transient stress. The distinction between an adaptive and a maladaptive response is, therefore, important: persistent glycolysis with suppressed oxidative phosphorylation, incomplete mitophagy, and sustained lipid peroxidation may create a feed-forward state in which the same dialysate exposure elicits progressively greater injury. Evidence from diabetic-kidney and chronic-kidney-disease models supports the relevance of mitochondrial dysfunction and glutathione depletion to this redox checkpoint [26,27,28]. The direction and magnitude of each pathway still require direct confirmation in human PD tissue.

### 3.3. Local and Systemic Metabolic Context

PD-associated metabolic stress is distributed across compartments. Dialysate reflects the local peritoneal microenvironment, whereas plasma reflects systemic glucose absorption, uremic toxins, inflammation, and nutritional status. Gut microbial metabolites provide an additional axis: in a cohort of patients receiving continuous ambulatory PD, microbiome structure, short-chain fatty acids, indole compounds, trimethylamine N-oxide, and barrier markers differed from healthy and non-dialyzed ESRD comparators [29,30]. A separate CKD cohort also linked gut-microbiota composition to inflammatory burden, supporting the need to record systemic covariates in effluent-metabolomics studies [31]. These data do not establish that gut-derived metabolites cause peritoneal fibrosis, but they support a systems view in which local dialysate metabolism is embedded in a broader uremic and nutritional network.

## 4. Metabolic Pathway Remodeling During Fibrotic Transformation

### 4.1. Glycolytic Remodeling and the HIF-1α–TGF-β Axis

Where substrate use has been assessed directly, a glycolytic switch can be defined by increased glucose uptake, diversion of pyruvate away from mitochondrial oxidation, lactate production, and coordinated activation of glycolytic enzymes. In mesothelial cells, inhibition of hyperglycolysis reduces fibrotic remodeling, providing direct evidence that glycolytic flux is functionally relevant rather than a passive correlate [6]. Pharmacological studies of canagliflozin and empagliflozin suggest that modulating glucose transport or senescence-associated signaling can reduce high-glucose-induced MMT in experimental systems [15,32]. A PD-focused review frames SGLT2 inhibition as a balance between glucose exposure, peritoneal effects, residual kidney function, and hemodynamic safety [33]. These findings are hypothesis-generating for PD because systemic sodium–glucose cotransporter 2 inhibition may have effects that are distinct from direct peritoneal actions.

HIF-1α integrates oxygen availability, redox state, and substrate flux. Stabilized HIF-1α promotes glycolytic genes, lactate production, and angiogenic mediators, while STAT3 can reinforce HIF-1α activity and inflammatory transcription. The STAT3/HIF-1α pathway has been implicated in high-glucose-induced PF, and IL-6 trans-signaling provides a plausible inflammatory bridge to STAT3-dependent structural change [8,16]. O-GlcNAcylation and pseudohypoxia may further connect glucose availability to HIF-1α activity and MMT [17,34]. In parallel, experimental inhibition of EZH2 has been reported to reduce peritoneal angiogenesis through a VEGFR2/ERK1/2/HIF-1α-dependent mechanism, reinforcing the link between metabolic oxygen sensing and vascular remodeling [35]. A testable hypothesis is that serial increases in lactate or lactate-derived ratios in dialysate may precede changes in CA-125, transport kinetics, or imaging/histological markers; this requires prospective validation.

### 4.2. Mitochondrial Dysfunction, ROS, and Mitophagy

Mitochondria are both energy-producing organelles and signaling platforms. PD-associated stress can lower ATP production and membrane potential, increase mitochondrial ROS, damage mitochondrial DNA, and impair fatty-acid oxidation. Mitochondrial injury then activates redox-sensitive inflammatory pathways, including NLRP3 inflammasomes, and releases mtDNA or other DAMPs. In kidney-fibrosis models, HIF-1α-BNIP3-mediated mitophagy limits mitochondrial ROS and NLRP3 activation, illustrating how mitochondrial quality control can restrain fibrotic signaling [36]. Mitophagy also interacts with ferroptosis through the ROS/HO-1/GPX4 axis [37]. Peritoneal studies of inflammasome activation and mitochondrial injury point in the same direction, although the relative contribution of each organelle and cell type remains unresolved [38,39]. Outside PD, a recent narrative review of cardiometabolic disease describes a linked, hypothesis-generating framework involving mitochondrial redox disruption, impaired antioxidant defenses, and ferroptosis; it provides mechanistic context but does not establish that a gut–mitochondria–ferroptosis sequence operates in PD-associated PF [40].

Direct PD experiments support the same biological direction. High-dialysate-glucose exposure is associated with oxidative stress and mitochondrial-mediated injury, while interventions that preserve mitochondrial function or reduce ROS can attenuate experimental PF [41,42]. The recent report that α-Klotho modulates BNIP3-mediated mitophagy and mitochondrial ROS in kidney injury provides a mechanistic template, but its translation to the peritoneum requires validation in mesothelial cells and human effluent [43]. Mitochonic acid-5 and autophagy-modulating strategies have also shown protection in experimental PF, supporting mitochondrial quality control as a testable intervention rather than a purely descriptive pathway [39,44]. A useful experimental panel should, therefore, measure oxygen consumption, extracellular acidification, ATP, membrane potential, mitochondrial ROS, mtDNA release, mitophagic flux, and NLRP3 activation together.

### 4.3. Lipid Metabolism, Fatty-Acid Oxidation, and Ferroptosis

Lipid metabolism provides a bridge between energy failure and regulated cell death. Mesothelial cells require flexible fatty-acid uptake and oxidation to maintain membrane composition and mitochondrial energy production. Restoration of CPT1A-mediated fatty-acid oxidation protects mesothelial cells from experimental PF, directly linking lipid flux to peritoneal remodeling [45]. Experimental and clinical work in diabetic kidney disease further indicates that impaired carnitine-dependent fatty-acid oxidation can lead to ectopic lipid accumulation, inflammation, and mitochondrial dysfunction, although this is mechanistic context rather than direct PD proof [46].

Ferroptosis is an iron-dependent form of regulated cell death characterized by phospholipid peroxidation and failure of the cystine–glutathione–GPX4 defense system. In PD-associated PF, it remains an emerging mechanism rather than an established human pathway. The proposed PD pathway is: impaired fatty-acid oxidation and iron handling increase oxidizable phospholipids; mitochondrial ROS and carbonyl stress amplify lipid peroxidation; SLC7A11-mediated cystine uptake, glutathione synthesis, or GPX4 activity becomes insufficient; and ferroptotic injury releases DAMPs that intensify inflammation and fibrosis. Mitophagy–ferroptosis coupling through ROS, HO-1, and GPX4 is well established in kidney injury models [37,47,48]. Lipophagy and the ESRRA axis have recently been implicated in controlling ferroptosis in kidney injury, providing additional nodes for testing in the peritoneum [49,50].

Several recent studies provide direct or near-direct experimental peritoneal evidence. Inhibition of PI3K/AKT/mTOR signaling activates autophagy and suppresses experimental PF, and VDAC2-dependent ferroptosis signaling has been associated with the time course of peritoneal fibrosis [42,51]. Quercetin was reported to increase GPX4 and SLC7A11 in a PD-associated PF model, while astragaloside IV and astragalus polysaccharide were linked to STAT3 or Nrf2/SLC7A11 regulation in peritoneal mesothelial injury [52,53,54]. Clinical iron-status measurements in PD patients treated with ferric citrate provide a feasible route for relating iron exposure to lipid-redox phenotypes, but they do not by themselves establish ferroptosis [55]. These results are promising but remain mostly preclinical; ferroptosis inhibitors should not be advanced clinically without demonstrating target engagement, tissue selectivity, and absence of adverse effects on host defense. Decreased GPX4 or SLC7A11 and increased oxidative stress are insufficient, by themselves, to establish ferroptosis. A convincing claim should combine iron dependence, oxidized-phospholipid or lipid-peroxidation measurements, impairment of the cystine–GSH–GPX4 system, compatible cellular or mitochondrial changes, pathway perturbation, and rescue by a ferroptosis-selective intervention. The current PD literature often provides only a subset of these criteria; natural products and multi-target agents should, therefore, be described as ferroptosis-related or ferroptosis-modulating unless the full evidence set is available.

### 4.4. Amino-Acid Metabolism and TCA-Cycle Anaplerosis

Amino acids support antioxidant synthesis, nitrogen handling, neurotransmitter and polyamine metabolism, and TCA-cycle anaplerosis. Glutamine can be converted to glutamate and α-ketoglutarate, replenishing the TCA cycle and supporting biosynthetic demands in activated fibroblasts. Glutamate and cysteine availability also controls glutathione synthesis and, therefore, ferroptosis sensitivity. In methylglyoxal-induced peritoneal injury, glutathione, glutamate, and ornithine metabolism are altered, supporting a direct connection between carbonyl stress and amino-acid redox metabolism [18]. The importance of this axis is consistent with work on glutathione restoration in CKD and with studies of arginine/NO metabolism in renal disease [28,56].

Targeted metabolomics of PD effluent has shown that alanyl-glutamine supplementation changes anti-oxidative metabolite patterns, and an experimental local alanyl-glutamine intervention has been proposed to resolve peritoneal immunometabolic dysfunction [57,58]. Arginine supplementation has likewise been reported to protect a gastrointestinal barrier exposed to long-term PD, although the study did not test peritoneal fibrosis directly [59]. These observations suggest that amino-acid therapy should be evaluated as a metabolic intervention rather than as nonspecific nutritional support. Key uncertainties include dose, intraperitoneal exposure, systemic absorption, acid–base effects, and whether altered effluent metabolites are causes, consequences, or pharmacodynamic markers.

### 4.5. Immunometabolism and Macrophage Plasticity

Macrophages respond to nutrient availability, lactate, lipid mediators, ROS, and cytokines. A glycolysis-dominant state can support rapid inflammatory transcription, whereas oxidative and lipid-associated programs may accompany repair or persistent profibrotic signaling. The binary M1/M2 model is, therefore, insufficient for chronic PD, in which macrophage states are plastic and shaped by repeated dialysate exposure. Peritoneal macrophage heterogeneity, inflammatory macrophage-to-CCL17-expressing transitions, and macrophage–mesothelial cross-talk have been linked to membrane dysfunction and PF [60,61]. Recent reviews emphasize that these cells are central drivers and therapeutic targets in dialysis-induced PF, while cautioning that macrophage markers do not map one-to-one onto metabolic flux [62,63]. Throughout this review, phenotypic polarization denotes marker or cytokine changes, metabolic association denotes pathway-level inference, and metabolic reprogramming is used only for studies that directly measure substrate flux, metabolite turnover, or coordinated metabolic enzyme activity. The broader term metabolic remodeling is used for the full evidence spectrum.

Recent studies connect metabolism to this cellular plasticity. PPARα activation by pemafibrate reduced inflammation, NLRP3 activation, and PF in a methylglyoxal model, suggesting that lipid sensing can reshape macrophage and mesothelial responses [64]. Deletion of macrophage p38 MAPK, modulation of macrophage extracellular traps, and targeting macrophage-associated signaling pathways each reduced experimental peritoneal injury, although the metabolic consequences were not always measured directly [65,66]. RIPK3-driven NLRP3/caspase-1/IL-1β signaling, PU.1-linked oxidative and inflammatory responses, and lncRNA-associated M2 polarization have also been implicated in experimental PF [67,68,69]. HDAC8 inhibition has been reported to reduce both MMT-related changes and M2-associated signaling, but the metabolic flux underlying this effect was not directly measured [70]. This distinction matters: a treatment may reduce fibrosis through immunosuppression without correcting the initiating metabolic defect. Future studies should combine single-cell transcriptomics with metabolite tracing and functional flux assays in sorted macrophage states.

## 5. Metabolism-Driven Cellular Communication

### 5.1. Mesothelial Cells: From Barrier Cells to Metabolic Sensors

Mesothelial cells form the first cellular interface with dialysate and coordinate barrier repair, innate immunity, and matrix homeostasis. High glucose, GDPs, ROS, and hypertonicity can reduce junctional integrity and induce MMT, a spectrum of phenotypic changes that includes loss of epithelial markers, acquisition of mesenchymal markers, and increased secretion of TGF-β, fibronectin, and collagen. We use MMT for this mesothelial-specific phenotypic transition; EMT is retained only when used by the cited source or when discussing a broader epithelial program. Reported pathway features include increased glycolytic demand, lactate accumulation, impaired mitochondrial reserve, and reduced metabolic flexibility; concentration or protein-expression changes alone are described as metabolic associations unless flux, turnover, or coordinated enzyme activity was measured. The relationship between MMT and metabolism may be bidirectional: metabolic stress can induce MMT, while the mesenchymal state can further alter substrate use and redox buffering [3,34,71].

Experimental work also links L-cysteine–PKM2 and Cyclo(His-Pro)–HDAC3 signaling to the injury-to-MMT transition; both findings remain preclinical [71,72].

The distinction between true transdifferentiation and a transient repair state is important. Effluent-derived mesothelial cells are heterogeneous and may include injured, activated, or shed cells; their transcriptome and metabolome cannot be interpreted as a direct biopsy of the entire membrane. Nevertheless, serial effluent cell and metabolite measurements could provide a minimally invasive window into the direction of cellular remodeling. Combining lactate, lipid-peroxidation products, mitochondrial-DNA fragments, CA-125, and mesothelial-cell phenotyping may improve biological specificity over any single marker.

### 5.2. Macrophages and Metabolite-Mediated Feedback

Macrophages receive signals from damaged mesothelial cells and, in turn, release cytokines, chemokines, ROS, proteases, and extracellular vesicles. MMP-10 deficiency reduced peritoneal inflammation and fibrosis through NF-κB pathway inhibition and was associated with lower transport abnormalities, demonstrating that macrophage and mesothelial signaling can be linked to a measurable functional endpoint [73]. CX3CL1–CX3CR1 interactions provide another route for macrophage–mesothelial cross-talk [61]. Metabolites such as lactate, succinate, prostaglandins, leukotrienes, and oxidized phospholipids may act as intermediate signals, but their concentrations, cellular sources, and receptor targets need to be resolved in vivo.

Macrophage-derived extracellular vesicles are especially relevant because they can transport proteins, lipids, nucleic acids, and metabolic enzymes. Exosome-based approaches have been reported to reverse or attenuate experimental PF, and macrophage extracellular traps have been implicated in fibrotic amplification [66,74,75]. Pediatric data also identify neutrophil extracellular traps as contributors to peritoneal inflammation and tissue remodeling, suggesting that extracellular-trap biology extends beyond macrophages [76]. These findings motivate a metabolite-plus-vesicle framework, but therapeutic vesicles require rigorous characterization of dose, biodistribution, cargo heterogeneity, and immunogenicity.

### 5.3. Fibroblasts, Pericytes, and Matrix-Building Metabolism

Fibroblasts are not passive recipients of TGF-β. Activation requires ATP, nucleotide and amino-acid supply, membrane synthesis, and collagen-processing capacity. Increased glycolysis can supply rapid ATP and biosynthetic intermediates, whereas glutaminolysis and α-ketoglutarate support TCA-cycle anaplerosis and epigenetic regulation. Single-cell studies have identified fibroblast subpopulations and peritoneal microenvironmental changes that are not captured by a single α-smooth muscle actin (α-SMA) marker [77,78,79]. A complementary single-cell analysis of mesenchymal-stem-cell and macrophage interactions further supports a multicellular rather than single-lineage model of membrane injury [80].

The transition from pericyte or other mesenchymal populations to myofibroblasts is an additional source of matrix-producing cells. TGF-β-dependent lymphangiogenesis and HIF-1α-linked angiogenic programs may further alter nutrient and oxygen delivery during remodeling [35,81]. Because collagen synthesis is energetically and metabolically demanding, the fibroblast metabolic phenotype may determine whether inflammation resolves or progresses to fixed membrane thickening. This hypothesis can be tested by integrating single-cell RNA sequencing, stable-isotope tracing of glucose or glutamine, and spatial metabolomics in serial experimental PF.

### 5.4. Extracellular Vesicles as Metabolic Couriers

Extracellular vesicles (EVs) provide a protected route for intercellular transfer of proteins, lipids, microRNAs, and metabolic regulators. Proteomic profiling of PD effluent-derived EVs has identified candidate signatures associated with membrane status, and a mouse model demonstrated that peritoneal EV cargo changes with fibrosis [82,83,84]. Mesothelial-cell EVs can promote fibroblast activation through integrin-linked kinase (ILK), illustrating how vesicles can convert a local metabolic injury signal into matrix remodeling [85].

The clinical value of EVs will depend on pre-analytical standardization. Dwell time, exchange volume, ultrafiltration, storage temperature, isolation method, and normalization to cell number or total protein all influence measured cargo. EV metabolomics and proteomics should, therefore, be reported with recovery controls and orthogonal validation, and should be analyzed alongside the soluble metabolome rather than as a replacement for it.

## 6. Metabolomics and Multi-Omics for Peritoneal Fibrosis

### 6.1. Specimen Choice and Analytical Platforms

Dialysate is an attractive matrix because it samples the local peritoneal environment repeatedly and without a biopsy. Plasma or serum provides systemic context, while effluent cells, EVs, and tissue (when clinically available) add cellular resolution. The main analytical platforms are liquid chromatography–mass spectrometry (LC–MS) for broad polar and lipid coverage, gas chromatography–mass spectrometry (GC–MS) for volatile or derivatized small molecules, and nuclear magnetic resonance (NMR) for robust, quantitative profiling with minimal sample preparation. A systematic review in *Metabolites* summarized the potential of metabolomics technologies for assessing peritoneal membrane profiles [11]. Mass spectrometry is also useful for characterizing non-enzymatic protein glycation and carbonyl-modified peptides, which could complement small-molecule GDP and AGE measurements [86].

Pre-analytical variables may dominate biological variance. Dwell duration, glucose concentration, icodextrin or amino-acid solution, peritonitis status, antibiotic exposure, residual kidney function, sampling time, fasting state, and storage delay should be recorded; dwell-related carbonyl stress can differ between solution types [87]. Effluent concentrations should be interpreted as mass-balance outputs rather than direct measures of local cellular production. Starting dialysate composition, plasma-to-peritoneal transport, membrane transport status, residual intraperitoneal volume, ultrafiltration, recovered effluent volume, and systemic metabolite concentration can each alter glucose, lactate, creatinine, amino-acid, and other small-molecule measurements. Studies should analyze matched unused dialysate and procedural blanks, quantify recovered volume, and, where feasible, report paired plasma concentrations, dialysate-to-plasma ratios, or net solute appearance.

For quantitative effluent studies, the primary estimand should be declared before analysis. If $C_{\mathrm{eff}}$ and $V_{\mathrm{rec}}$ denote the measured effluent concentration and recovered volume, and $C_{\mathrm{start}}$ and $V_{\mathrm{fill}}$ denote the concentration and volume of the instilled solution, the first-order apparent net change in solute recovered from the cavity is $A_{\mathrm{app},\mathrm{rec}}=C_{\mathrm{eff}}V_{\mathrm{rec}}-C_{\mathrm{start}}V_{\mathrm{fill}}$. This is a compartment-level balance, not a measure of production by peritoneal tissue: plasma-to-cavity transport, cellular release or uptake, absorption, chemical degradation, ultrafiltration/convection, and incomplete recovery can all contribute. If residual fluid is present, a complete two-compartment balance should include the residual compartment at both ends of the dwell, for example $\Delta M_{\mathrm{cavity}}=C_{\mathrm{eff}}V_{\mathrm{rec}}+C_{\mathrm{res},1}V_{\mathrm{res},1}-\left[C_{\mathrm{start}}V_{\mathrm{fill}}+C_{\mathrm{res},0}V_{\mathrm{res},0}\right]$, where subscripts 0 and 1 denote the beginning and end of the exchange. Estimating residual volume and its concentration requires direct measurements or an explicitly stated mixing assumption; when they are unavailable, the simpler expression should be labeled an apparent recovered-solute change and should not be interpreted as local secretion. We define “recovered-volume-normalized concentration” as $C_{\mathrm{rv}}=M_{\mathrm{rec}}/V_{\mathrm{rec}}$, where $M_{\mathrm{rec}}=C_{\mathrm{eff}}V_{\mathrm{rec}}$; for a well-mixed recovered sample, $C_{\mathrm{rv}}=C_{\mathrm{eff}}$ exactly and, therefore, this term is not an additional normalization. If a common reference volume $V_{\mathrm{ref}}$ is used, the scaled quantity $M_{\mathrm{rec}}/V_{\mathrm{ref}}$ should be reported as a reference-volume-scaled amount with $V_{\mathrm{ref}}$ stated explicitly, rather than relabeled as the measured effluent concentration. A dwell-time-normalized appearance rate ($A_{\mathrm{app},\mathrm{rec}}/t_{\mathrm{dwell}}$), total recovered amount, and raw $C_{\mathrm{eff}}$ can, therefore, be reported together. Matched plasma concentrations allow a dialysate-to-plasma ratio ($D/P=C_{\mathrm{eff}}/C_{\mathrm{plasma}}$); the ratio and net amount should be interpreted with ultrafiltration because convection can increase solute removal without local production. The 1.5%, 2.5%, and 4.25% glucose solutions differ in starting glucose, osmotic gradient, water transport, and recovered volume, so glucose strength, fill volume, dwell time, and ultrafiltration should be stratified or included as prespecified covariates rather than treated as interchangeable exposures. Sensitivity analyses should repeat the model using total amount, $A_{\mathrm{app},\mathrm{rec}}/t_{\mathrm{dwell}}$, D/P, and residual-volume-adjusted estimates, with plasma glucose, creatinine, urea, dialysis vintage, and transport status included as covariates. These steps reduce dilutional and convective bias but do not prove local cellular secretion.

Residual kidney function (RKF) is a time-varying confounder that deserves separate treatment. As dialysis vintage increases, declining urine volume and urea/creatinine clearance can change plasma concentrations, systemic substrate exposure, and the amount of a solute available to enter or leave the peritoneal cavity, even when local membrane production is unchanged. Consequently, an association between an effluent metabolite and PET transport or dialysis vintage in Table 1 may partly reflect RKF trajectory rather than local fibrosis. Candidate-signature studies should record serial urine volume and measured or estimated residual urea/creatinine clearance, align sampling to dialysis vintage and recent peritonitis, and use within-person or mixed-effects models that include RKF trajectory, plasma concentration, glucose exposure, and ultrafiltration. Adjustment is necessary but not sufficient for PF specificity: discrimination should be tested with a prespecified Tier I PF/EPS case definition, comparator groups with non-fibrotic membrane dysfunction or recent peritonitis, blinded structural adjudication, and independent external validation. Because the selected human effluent studies did not include these contrasts, they provide no direct evidence that adjustment alone distinguishes fibrosis from other membrane changes. A marker should be considered PF-specific only when its association persists after this adjustment and is reproduced against a structural or clinically adjudicated outcome.

Analytical reporting should distinguish unambiguous metabolite identification from putative annotation. Key candidates should be confirmed with authentic standards matched for retention time and tandem mass spectra. Matrix effects and ion suppression should be evaluated with matrix-matched recovery or post-extraction addition, using stable-isotope internal standards where available. Pooled quality-control samples, signal-drift monitoring, batch correction, and prespecified acceptance thresholds for analytical coefficients of variation should be reported. Authors should state whether results are absolute concentrations or relative signal intensities, how zero, missing, below-detection, and below-quantification values were handled, and how multiple testing was controlled. Creatinine, urea, total protein, sodium, and effluent volume may be evaluated as normalization covariates, but normalization should be prespecified and checked not to remove true biological signal. Inter-laboratory reproducibility and longitudinal within-person designs are preferable to single cross-sectional comparisons [11].

### 6.2. Candidate Metabolic Signatures

Beyond single-analyte lists, a stimulus-resolved metabolomics framework developed in aging skeletal muscle proposes pairing metabolite measurements with signaling context and the relevant biological challenge. This principle may inform PD study design, although the cited work concerns aging skeletal muscle rather than the peritoneum [88].

NMR-based profiling of overnight PD effluents from 125 patients discriminated high from low PET transport using alanine, creatinine, glucose, and lactate (reported composite AUC = 0.975) [89]. This result was single-cohort discrimination of PET transport categories, without external validation; it cannot be interpreted as fibrosis prediction, early PF detection, or evidence of histologically confirmed disease. In 118 PD-associated peritonitis episodes, effluent lactate was higher in episodes with adverse outcomes (8.50 [5.48–13.1] versus 5.40 [3.18–8.25] mmol/L), with an OR of 1.419 per reported model increment and an AUC of 0.752 [90]. These estimates concern acute infection and cannot be transferred directly to chronic PF prediction. Targeted profiling in 20 stable patients detected 184 of 188 predefined metabolites and showed that 8 mM alanyl-glutamine altered amino-acid and redox-related metabolites, supporting pharmacodynamic rather than PF-diagnostic use [57]. More recently, a prospective cohort of 117 patients linked high peritoneal inflammation to subsequent peritonitis (HR = 3.8; 95% CI, 1.8–8.2; p=0.0004), with 1019 metabolites analyzed and a separate multicenter validation cohort of 37 patients [58]. Together, these data demonstrate analytical and clinical-signal feasibility, but not a validated fibrosis-specific signature.

Potential candidate classes include: (i) glycolytic metabolites such as lactate and pyruvate; (ii) TCA-cycle metabolites such as citrate and succinate; (iii) glutamine, glutamate, ornithine, and arginine-related metabolites; (iv) lipid mediators, phospholipid oxidation products, and acylcarnitines; (v) carbonyl and AGE-related products; and (vi) redox markers linked to glutathione, thioredoxin, and NADPH. None should be considered a validated PF biomarker until replicated across solution types, dialysis vintages, ethnic groups, and clinically adjudicated outcomes.

The current human evidence is better described as a set of membrane-function, inflammatory-event, and pharmacodynamic signals than as a validated fibrosis biomarker panel. Table 1 summarizes representative human studies and makes sample size, reported quantitative metrics, clinical endpoint, and fibrosis status explicit. The reported AUC, odds ratio, hazard ratio, and microbiome discrimination statistics are study-specific and were not pooled because the populations, endpoints, and units are not commensurate.

The human studies in Table 1 do not establish a structural PF biomarker. Table 2, therefore, separately summarizes experimental studies that directly perturbed a metabolic pathway and measured a structural or functional peritoneal endpoint. Each row represents one primary study; exposure, sample size, and significance are reported when available in the source. “NR” denotes that the primary publication did not provide a directly reportable numerical value for that field; no value was inferred from figures or indirect descriptions.

**Table 1:** Selected narrative evidence map of representative human metabolomics and metabolite studies relevant to PD membrane injury. The table is not an exhaustive or systematically sampled inventory.

Study	Sample/Design	Platform	Candidate Metabolites	Reported Quantitative Result	Endpoint	PF?	Main Limitation
Asano et al. [3]	50 PD patients; PET	CE-TOF-MS	38 small molecules	Urea, citrulline, and choline had lower D/P than creatinine; 3 regression coefficients < 0.3 and 5 were negative	Transport (D/P creatinine)	No	Cross-sectional transport study; no histology or external validation.
Wiesenhofer et al. [57]	20 stable PD patients; crossover RCT	Targeted panel (188)	Alanine, glutamine, methionine sulfoxide, acylcarnitines	184/188 detected; 175 increased and 9 decreased over 4 h; hexoses −3.8-fold; 8 mM alanyl-glutamine changed redox metabolites	Pharmacodynamic response	No	Intervention-associated change; no structural PF endpoint.
Kim et al. [89]	125 patients; first PET	^1^H-NMR; OPLS-DA	Alanine, creatinine, glucose, lactate	Four-metabolite composite AUC = 0.975 for high vs. low PET transport	PET transport category	No	Single cohort; transport phenotype without histology or adjudicated PF.
Li et al. [29]	72 CAPD, 13 ESRD, 13 healthy; cross-sectional	16S rDNA, GC-MS, ELISA	SCFAs, indoles, TMAO, D-lactate, PCS	Microbiome LDA > 4; D-lactate, PCS, and TMAO differed from healthy controls (*p* < 0.05)	Dialysis vintage/systemic context	No	Indirect relation to the membrane; no structural PF assessment.
Yu et al. [90]	118 PDAP episodes/ 100 patients; retrospective	Lactate assay	Lactate	No-cure 8.50 [5.48–13.1] vs. cure 5.40 [3.18–8.25] mmol/L; OR = 1.419; AUC = 0.752; threshold 10.2 mmol/L	PDAP adverse outcomes	No	Acute infection signal; cannot establish chronic fibrosis.
Herzog et al. [58]	117 prospective patients; 207 PETs; validation n=37	Targeted panel (1019) plus proteomics	Glucose and amino-acid pathways; alanyl-glutamine response	HR = 3.8 (95% CI, 1.8–8.2; p=0.0004); 265/1019 metabolites quantifiable; 154 differed at p<0.05	Immune function/ peritonitis	No	Longitudinal with multicenter validation; no structural PF endpoint.

**Table 2:** Selected narrative evidence map with numerical primary-study extraction linking metabolic pathway perturbation to peritoneal remodeling. Each row represents one cited primary study; “NR” denotes a numerical field for which no directly reportable value was provided by the primary publication. The table is not an exhaustive inventory.

Study	Design/Sample	Exposure, Dose, and Time	Numerical Result	PF/Function Endpoint and Evidence Boundary
Si et al. [6]	Human mesothelial scRNA-seq, cultured mesothelial cells, and PDF mouse model; biological *n* NR	2-deoxyglucose and intraperitoneal AAV microRNA triad; dose and duration NR	Structural effect size, uncertainty, and exact *p* value NR; both interventions reduced experimental PF	Histological PF and MMT markers. Tier III causal evidence; the human component was association, not intervention.
Yang et al. [8]	MET-5A assays, n=3 experiments; C57BL/6J mice, n=5/group	60 mmol/L glucose for 48 h; S3I-201 10 μM in vitro; 4.25% PDF 100 mL/kg/day plus S3I-201 10 mg/kg/day for 28 days	S3I-201 reduced all principal cellular and in-vivo comparisons versus HG/PDF (p<0.05; several cellular assays p<0.01 or p<0.001); absolute effect sizes NR	Membrane thickening, collagen I, α-SMA, angiogenesis, and macrophage infiltration. Tier III; small groups and no human structural endpoint.
Si et al. [16]	PDF mice, n=6/group; human omentum, n=3 with ultrafiltration failure versus n=3 controls	4.25% PDF 100 mL/kg/day for 6 weeks; endothelial PFKFB3 deletion; 3PO 25 mg/kg three times/week	Endothelial PFKFB3 transcript level was >3-fold higher after PDF; genetic/pharmacological blockade improved structural and PET measures (p<0.05); exact effects NR	Thickness, capillary density, ultrafiltration, D/D_0_ glucose, and D/P urea. Tier III with small supportive human tissue series.
Su et al. [45]	Human effluent-derived cells, n=12 long-term PD versus n=6 controls; PDF mice, n=6/group	4.25% PDF 100 mL/kg/day for 6 weeks; C75 5 mg/kg or etomoxir 30 mg/kg three times/week	>79% of COL1A1^+^ cells localized to the mesothelial population; human CPT1A and animal structural/PET comparisons p<0.05; exact effects NR	Collagen deposition, D/D_0_ glucose, and D/P urea. Tier III bidirectional perturbation plus human association; compound specificity is limited.
Hirahara et al. [18]	Rat MGO/PDF metabolomics model; group *n* NR	Intraperitoneal MGO-containing PDF for 21 days; MGO concentration/dose NR	Fold changes and exact *p* values NR; methionine sulfoxide, GPX activity, glutathione, glutamate, aspartate, ornithine, and AGEs increased	Peritoneal thickening and mesenchymal-cell proliferation. Tier III exposure-response association without pathway-specific rescue.
Huang et al. [91]	CG model, n=7/group for MSC-EVs and n=5/group for GNE-140; PDF validation, n=5/group	0.1% CG three times/week for 3 weeks or 4.25% PDF 0.1 mL/g/day for 6 weeks; MSC-EVs or GNE-140 5 μg/g three times/week	Structural, inflammatory, and PET comparisons ranged from p<0.05 to p<0.001; exact effects NR; scRNA-seq included 11,543 cells from n=3/group	Thickness, FN1/α-SMA, D/D_0_ glucose, and D/P creatinine. Tier III; EV cargo heterogeneity and CG injury limit attribution.
Ruan et al. [34]	High-glucose mesothelial-cell and PDF mouse models; group *n* NR	GSDME deficiency or melatonin; dose and duration NR	Effect sizes, uncertainty, and exact *p* values NR; both interventions reduced fibrosis/angiogenesis and preserved ultrafiltration	Histological PF, angiogenesis, and ultrafiltration. Tier III; primarily pyroptosis/mitochondrial protection rather than metabolism-specific therapy.

Proteomics complements metabolomics by identifying enzymes, receptors, ECM proteins, and EV cargo. Earlier studies characterized peritoneal dialysate proteomes across membrane types and before/after dialysis [92,93,94]. Recent work has expanded to effluent-derived EVs and pediatric cohorts [95,96]. Because proteins and metabolites occupy different causal layers, integrative models should preserve their temporal order rather than concatenate all features into an opaque classifier.

### 6.3. The Spatial Sampling Gap and Tissue-Resolved Validation

Effluent is a valuable, repeatable window onto the luminal compartment, but it is not a biopsy of the whole peritoneal membrane. It preferentially captures soluble exometabolites, shed or detached mesothelial cells, leukocytes, and extracellular vesicles released into the cavity. It samples the submesothelial compact zone, vascular endothelium, pericytes, and deep interstitial myofibroblasts embedded in dense collagen much less completely. A low or absent effluent signal, therefore, cannot be taken as evidence that a pathway is inactive in the tissue compartment where fixed fibrosis is developing.

Matrix-assisted laser desorption/ionization mass-spectrometry imaging (MALDI-MSI) and related spatial metabolomics could address this blind spot by mapping metabolites, lipids, and oxidized phospholipids directly across tissue sections while preserving their anatomical coordinates. A practical spatial multi-omics validation design would use serial sections for MALDI-MSI, spatial transcriptomics or proteomics, and multiplex histology, with co-registration to collagen, endothelial, pericyte, mesothelial, and myofibroblast markers. Tissue handling, matrix effects, ion suppression, spatial resolution, and analyte identification should be controlled with matrix-matched and isotope-labeled standards; these methods should be treated as a proposed validation route rather than as an established clinical test.

Spatial measurements can make the effluent–tissue relationship testable. Regions of interest could compare relatively avascular fibrotic patches with hypervascularized granulation tissue and quantify local gradients in lactate/pyruvate, succinate/citrate, acylcarnitines, glutathione-related species, and lipid-peroxidation products. Co-localizing these maps with oxygenation or pseudohypoxia markers, vessel density, collagen architecture, and spatially resolved inflammatory and fibroblast states would distinguish a deep hypoxic/redox niche from a high-flow inflammatory niche. Paired effluent and tissue sampling could then ask which spatial signals spill into the cavity, which remain compartmentalized, and whether a candidate effluent marker tracks regional remodeling after adjustment for dwell, ultrafiltration, and RKF.

### 6.4. From Association to a Proposed Metabolic Fibrosis Atlas

The proposed metabolic fibrosis atlas would connect metabolites to enzymes, cell states, pathways, and clinical variables. A practical architecture is a three-layer network: a metabolite layer (measured concentrations and isotopologues), a cellular layer (mesothelial, macrophage, fibroblast, endothelial, and pericyte states), and a clinical layer (transport, ultrafiltration, peritonitis, residual kidney function, and membrane histology). Single-cell transcriptomics can define cell states, but transcript abundance does not establish metabolic flux; targeted metabolomics, enzyme activity, and isotope tracing are needed for functional validation. This is a proposed framework rather than an established atlas.

**Figure 1:**
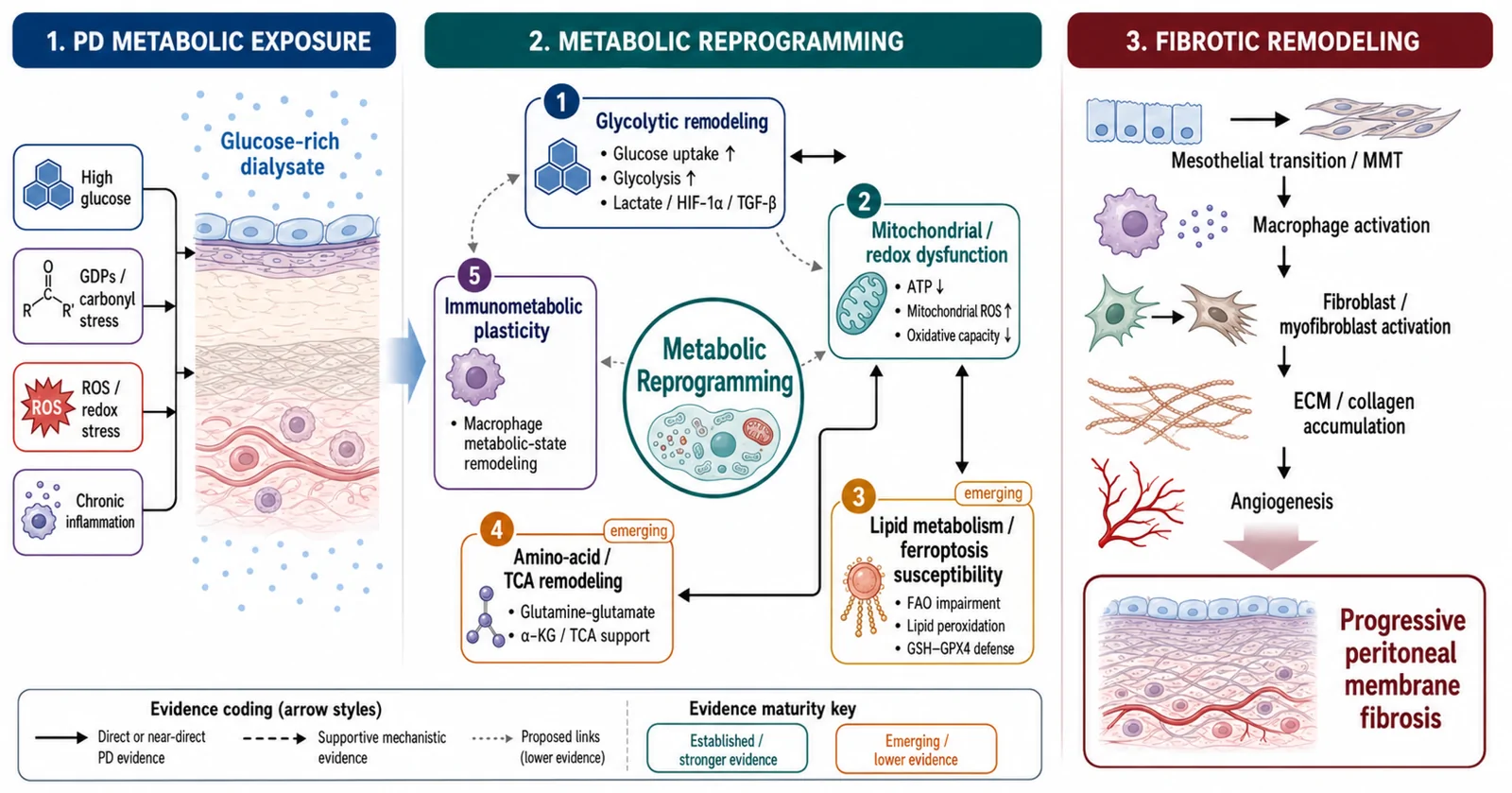
Metabolism-centered landscape in PD-associated peritoneal fibrosis. Chronic exposure to glucose-rich dialysate, GDPs, ROS, and inflammatory signals converges on glucose, mitochondrial, lipid/ferroptosis, amino-acid, and immunometabolic modules. These modules are proposed to converge on MMT, fibroblast activation, angiogenesis, ECM deposition, and progressive membrane fibrosis; the relative contribution of each module remains evidence-tier dependent. Abbreviations: GDP, glucose degradation product; ROS, reactive oxygen species; MMT, mesothelial-to-mesenchymal transition; ECM, extracellular matrix.

Machine learning can assist feature selection and risk prediction, but small cohorts, batch effects, missing data, and leakage from repeated samples can inflate performance. Candidate models should be locked before external validation, report calibration as well as discrimination, and compare against simple clinical baselines. Explainable models that nominate a small, biologically coherent panel are more likely to translate than high-dimensional signatures without a mechanistic anchor. Figure 1 summarizes the full metabolism-to-fibrosis framework.

**Figure 2:**
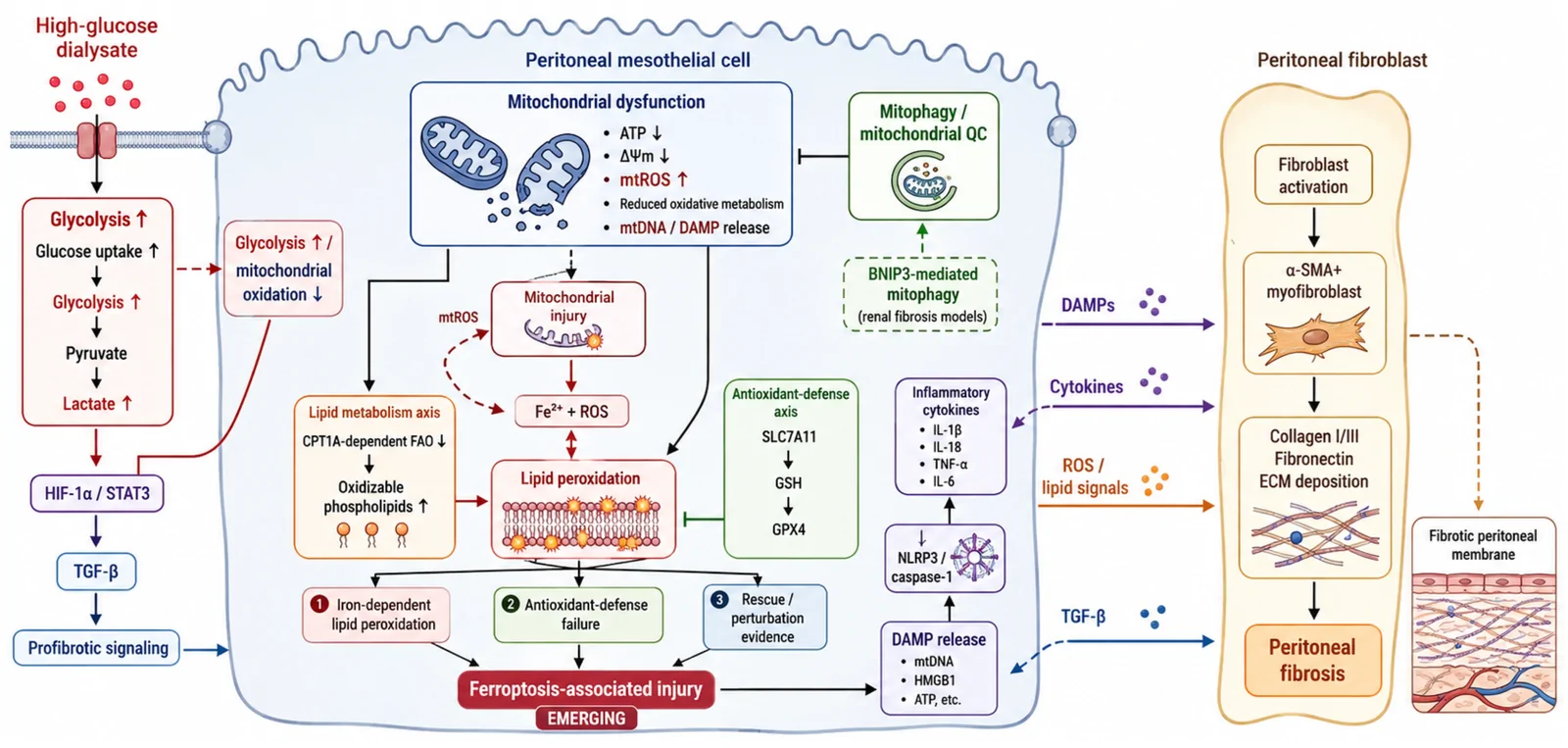
Proposed glycolysis–mitochondria–ferroptosis crosstalk. High-glucose stress is associated with increased glycolysis and lactate, while mitochondrial ROS, NLRP3 activation, lipid peroxidation, and ferroptosis-related injury are supported by a mixture of direct PD and extrapolated renal-model evidence. DAMPs and profibrotic mediators may then amplify fibroblast activation. Directional arrows denote hypotheses and reported associations rather than a validated linear pathway. Abbreviations: ROS, reactive oxygen species; NLRP3, NOD-like receptor family pyrin domain-containing 3; DAMP, damage-associated molecular pattern; GPX4, glutathione peroxidase 4.

Figure 2 separates PD-supported links from renal-model extrapolation within the glycolysis–mitochondria–ferroptosis axis.

## 7. Therapeutic Opportunities and Translational Priorities

Therapeutic opportunities are organized by study design, endpoint, and clinical readiness rather than presented as equivalent options. Ferroptosis-directed studies are evaluated first in Table 3 because their interpretation requires orthogonal mechanistic criteria.

### 7.1. Dialysate Engineering and Exposure Reduction

Biocompatibility remains the most direct metabolic intervention. Neutral-pH, bicarbonate or bicarbonate/lactate-buffered solutions, reduced GDP content, alternative osmotic agents, and tailored use of icodextrin or amino-acid solutions can lower chemical and carbonyl stress. Reviews and clinical studies report improved cellular compatibility and more favorable long-term transport profiles with newer formulations [21,97,98]. Effects on hard clinical outcomes and fibrosis progression are not uniform, and recent narrative reviews emphasize the need to individualize fluid selection [4,99]. Fluid choice should be viewed as a metabolic exposure decision that also influences systemic glucose load, nutrition, acid–base balance, and lipid metabolism.

### 7.2. Glycolysis and Mitochondrial Protection

The hyperglycolysis data support a strategy of normalizing flux rather than completely blocking glycolysis. Candidate interventions include improving glucose handling, reducing HIF-1α/STAT3 overactivation, restoring pyruvate entry into mitochondria, and preserving NADPH and glutathione regeneration. Mitochondrial strategies include antioxidants targeted to mitochondria, enhancement of mitophagy when flux is impaired, and protection of CPT1A-dependent fatty-acid oxidation. Hydrogen, rapamycin, and other experimental agents have reduced PF in models through ROS, AKT/mTOR, or lipid-homeostasis pathways [45,100,101]. Gasotransmitters and mitochondria-directed agents are additional mechanistic probes, but their pharmacokinetics and safety in PD remain unknown [39,102].

### 7.3. Lipid Redox and Emerging Ferroptosis-Related Targets

Ferroptosis-oriented therapy should be guided by tissue or effluent evidence of target engagement. Potential approaches include restoring cystine uptake and GPX4 activity, limiting iron-catalyzed lipid oxidation, correcting carnitine or fatty-acid oxidation defects, and modulating Nrf2/SLC7A11 signaling. Quercetin, astragaloside IV, and astragalus polysaccharide are useful preclinical probes, but their effects may reflect broader antioxidant or anti-inflammatory actions rather than selective ferroptosis inhibition [52,53,54]. A rigorous trial would require pre-specified metabolite and lipid-oxidation endpoints, an active comparator, and monitoring for infection or impaired immune surveillance.

### 7.4. Immunometabolic and Bioactive-Compound Strategies

A recent gut–liver–brain axis perspective discusses how dietary exposures may influence systemic metabolic and inflammatory networks; this provides a conceptual rationale for nutrition-sensitive studies but no direct evidence of benefit in PD-associated PF [103]. Within PD/PF models, PPARα, macrophage p38, inflammasome, STING, and EV pathways provide opportunities to uncouple persistent inflammation from metabolic injury. PPARα activation with pemafibrate, macrophage-modulating cell therapies, and EV-based interventions have each shown protective effects in experimental PF [64,65,104]. A separate EV-based study provides convergent evidence but also highlights the need for standardized cargo and dosing [91]. Chemically defined bioactive compounds, including zinc-related strategies, oligo-fucoidan, resveratrol glycosides, and micheliolide, should be treated as hypothesis-generating tools rather than clinically established therapies [75,105,106]. Their future evaluation should use metabolomics to identify active exposure-response relationships and should distinguish direct mesothelial effects from systemic nutritional or immunological effects [107].

Because GPX4 or SLC7A11 expression alone does not establish ferroptosis, Table 3 evaluates each direct PD/peritoneal study against orthogonal criteria. The table distinguishes a ferroptosis-selective rescue from general antioxidant or anti-inflammatory improvement.

**Table 3:** Selected narrative evidence map with numerical primary-study appraisal of emerging ferroptosis evidence in PD-associated peritoneal injury. Each row represents one cited primary study; “NR” denotes a numerical field for which no directly reportable value was provided by the primary publication. The table is not an exhaustive inventory.

Study	Model, Sample, and Exposure	Numerical Ferroptosis Readouts	Rescue/Perturbation	Structural/Functional Result and Evidence Boundary
Wei et al. [51]	Erastin-treated MET-5A cells and mouse PF; erastin/Fer-1 doses, group *n*, and duration NR	Tissue iron, ROS, lipid peroxidation, ACSL4, and VDAC2 increased; GSH decreased; numerical effects and *p* values NR; chelator test NR	Fer-1 reversed cellular and in-vivo injury; VDAC2 siRNA plus NAC gave additional rescue; numerical rescue effect NR	Collagen deposition, dysfunction, and pathology improved with Fer-1. Among the more direct PD-oriented studies in this set, but erastin is an induced-ferroptosis model.
Ouyang et al. [52]	Effluent cells stratified by PD duration, high-glucose MET-5A cells, and rat PF; group *n*, quercetin dose, and duration NR	GPX4 and SLC7A11 decreased; exact changes and *p* values NR; direct iron-dependence and oxidized-lipid quantification NR	Fer-1 was the in-vitro positive comparator; quercetin restored GPX4/SLC7A11 to a comparable direction; between-treatment effect NR	Peritoneal thickness/fibrosis effect NR. Human evidence is marker association; multi-target quercetin does not establish selective rescue.
Liu et al. [53]	MGO/PDF mice, n=5/group allocation (histology n=3, immunoblot n=4); HMrSV5 assays, n=3; 40 mM MGO 100 mL/kg/day or 4.25% PDF for 4 weeks; AS-IV 15 mg/kg; S3I-201 10 mg/kg; cells: 3% PDF for 24 h	ROS, Fe^2+^, lipid peroxide, MDA, GPX4, and mitophagy markers changed with injury and rescue (p<0.05 to p<0.001); direct iron-chelation test NR	NAC 5 mM, Mdivi-1 10 μM, liproxstatin-1 200 nM, S3I-201 100 μM, and AS-IV 200 μM were mechanistic comparators	AS-IV improved histopathology and oxidative/ferroptosis readouts; structural effect size NR. Supports STAT3-linked mitophagy/ferroptosis, with natural-product pleiotropy.
You et al. [54]	PDS/erastin-treated mesothelial cells and PDS/sorafenib mouse model; group *n*, APS dose, and duration NR	Fe^2+^, ROS, lipid peroxide, and MDA increased; GSH, Nrf2, SLC7A11, and GPX4 decreased; effect sizes and *p* values NR	Fer-1 positive control; APS produced dose-dependent partial rescue; exact between-treatment effect NR	In-vivo ferroptosis-related measures and PF improved; effect size NR. Orthogonal criteria are present, but APS is multi-target and no human validation was reported.

Table 4 orders representative intervention studies by translational readiness: (i) clinically implemented dialysis-fluid strategies, (ii) human exploratory or pharmacodynamic interventions, and (iii) preclinical mechanistic candidates. These categories reflect current human use and endpoint directness, not comparative efficacy.

**Table 4:** Selected narrative evidence map with numerical primary-study extraction for metabolic and immunometabolic therapeutic strategies relevant to PD-associated peritoneal injury, ordered from clinically implemented strategies to human exploratory interventions and preclinical candidates. Each row represents one cited primary study. “NR” indicates that the primary publication did not provide a directly reportable numerical effect size. The table is not an exhaustive inventory.

Study	Design/Sample	Dose/Exposure and Time	Target	Numerical Study-Level Result	Human Evidence, Readiness, and Boundary
Elphick et al. [98]	Prospective multinational cohort, n=366; 71 consistently used biocompatible solutions	Biocompatible versus standard glucose-based solutions during longitudinal PET follow-up	Reduced GDP/pH exposure	Adjusted D/P creatinine was 0.67 versus 0.72 (p=0.02). Peritonitis-associated D/P change was +0.020 (95% CI, 0.01–0.03) per episode with standard solution versus −0.014 (95% CI, −0.03 to <0.01) with biocompatible solution	Direct human cohort. Clinically available; transport is a functional surrogate, not a structural PF endpoint, and exposure was not randomized.
Wiesenhofer et al. [57]	Randomized crossover, n=20 stable PD patients	3.86% glucose PDF with 8 mM alanyl-glutamine; 4-h PET; 28–35-day washout	Amino-acid/redox pharmacodynamics	184/188 metabolites detected; 175 increased and 9 decreased over the 4-h dwell; hexoses decreased 3.8-fold; assay correlations: creatinine r=0.968, glucose r=0.844; intervention effect sizes NR	Direct human intervention. Analytical validity and target engagement are shown; no longitudinal membrane-function or structural PF outcome.
Herzog et al. [58]	Prospective cohort n=117 (207 PETs), short-term crossover n=20, multicenter phase-2 RCT analysis n=37	3.86% PDF; local 8 mM alanyl-glutamine; RCT periods 8 weeks each	Peritoneal immune metabolism	HR = 3.8 (95% CI, 1.8–8.2; p=0.0004; mean follow-up 552 days); 265/1019 metabolites quantifiable, 154 altered at p<0.05 and 136 at q<0.05	Direct human evidence with validation. Endpoint was peritonitis/immune function, not histological PF.
Yang et al. [8]	MET-5A assays, n=3; PDF-exposed mice, n=5/group	Cells: 60 mmol/L glucose for 48 h plus S3I-201 10 μM; mice: 4.25% PDF 100 mL/kg/day plus S3I-201 10 mg/kg/day for 28 days	STAT3; HIF-1α glycolytic signaling	Principal cellular and in-vivo comparisons p<0.05; several cellular assays p<0.01 or p<0.001; absolute structural effects NR	No human intervention. PD-directed Tier III evidence; small groups and no human pharmacokinetic or safety data.
Wang et al. [15]	HMrSV5 cells and 4.25% PDF-exposed rats, n=6/group, 5 weeks	Canagliflozin 15 μM in vitro and 10 mg/kg/day intraperitoneally in vivo	HIF-1α; TGF-β/Smad3; glucose transport	Reduced peritoneal thickness and fibrotic proteins and improved glucose absorption, D/P creatinine, and ultrafiltration (p<0.05 or p<0.001 across reported comparisons)	No human intervention. Preclinical local dosing; SGLT2-independent and off-target effects remain possible.
Su et al. [45]	PDF mice, n=6/group; human effluent cells, n=12 long-term PD versus n=6 controls	4.25% PDF 100 mL/kg/day for 6 weeks; C75 5 mg/kg or etomoxir 30 mg/kg three times/week	CPT1A-mediated FAO	>79% of COL1A1^+^ cells were mesothelial; C75 improved collagen/PET and etomoxir worsened both (p<0.05); exact effects NR	Human association only. Bidirectional perturbation supports target relevance, but both compounds have pharmacological limitations.
Jia et al. [42]	Rat PMCs and 4.25% PDF/LPS rat model, 4 weeks; quantitative in-vivo assays n=3/group	LY294002 or rapamycin 100 μM/kg/day from week 2; LPS 0.6 mg/kg on days 1, 3, 5, and 7	PI3K–AKT–mTOR; autophagy; redox balance	Both agents reduced ROS/MDA and α-SMA/FSP1 and restored GSH/GPX and epithelial markers; reported comparisons p<0.05 to p<0.01	No human evidence. Very small quantitative animal groups; the reported dose unit is unusual, and membrane function was not assessed.
Huang et al. [91]	CG model: MSC-EVs n=7/group, GNE-140 n=5/group; PDF validation n=5/group; scRNA-seq 11,543 cells from n=3/group	0.1% CG three times/week for 3 weeks or 4.25% PDF 0.1 mL/g/day for 6 weeks; MSC-EVs/GNE-140 5 μg/g three times/week	LDHA–lactate; H3K18 lactylation; CCL2	Thickness, FN1/α-SMA, inflammatory cells, D/D_0_ glucose, and D/P creatinine improved (p<0.05 to p<0.001); exact effects NR	No human intervention. Convergent EV/enzyme-inhibitor evidence; EV cargo heterogeneity and CG injury limit attribution.
Shinkai et al. [64]	MGO mouse model, n=5/group; 40 mM MGO, 2.5 mL, 5 days/week for 3 weeks	Pemafibrate 0.3 mg/kg/day in diet, started 3 weeks before MGO	PPARα; NLRP3; inflammatory metabolism	Reduced peritoneal thickness, collagen I/III, α-SMA/FSP1, and abnormal D/P urea and D/D_0_ glucose (p<0.05)	No human intervention. Preventive pre-exposure design; metabolic flux and human PF efficacy remain untested.
Ikushima et al. [65]	CG-treated macrophage-specific p38-MAPK-knockout mice versus CG controls, n=11 versus 11	0.1% CG 0.01 mL/g three times/week for 3 weeks; macrophage-specific p38 deletion	Macrophage inflammatory signaling	Mapk14 mRNA decreased 35%; membrane thickness 280.6 to 160.7 μm (43% reduction); fibrotic/inflammatory comparisons p<0.05 or p<0.01	Human tissue association only. Strong cell-specific target validation, but not a drug intervention or direct metabolic-flux evidence.

**Figure 3:**
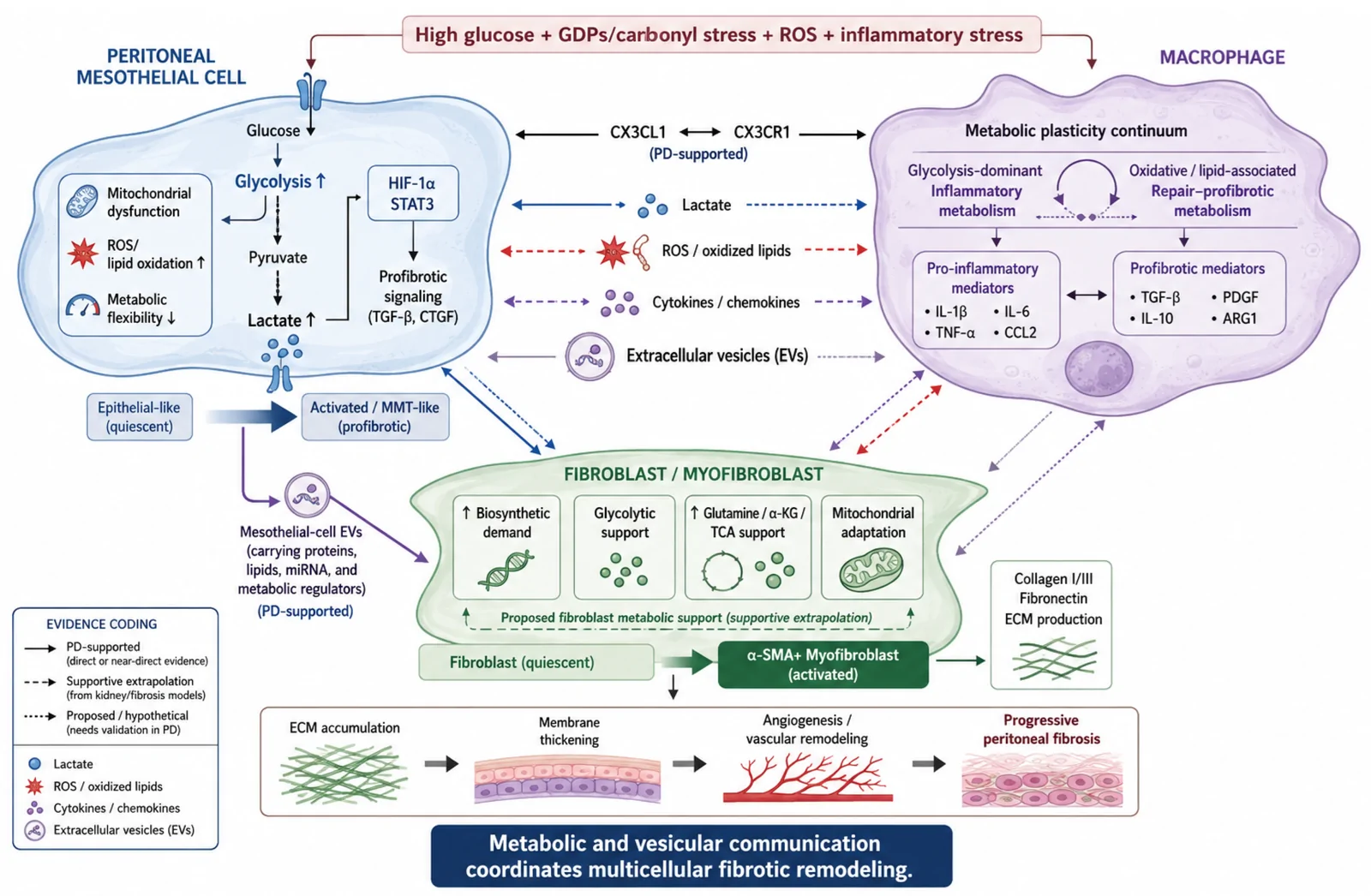
Proposed metabolic communication among mesothelial cells, macrophages, and fibroblasts. Metabolites, ROS, lipid mediators, cytokines, and EVs are candidate mediators of bidirectional feedback that may coordinate MMT, immune-cell plasticity, fibroblast activation, and tissue-level remodeling. Abbreviations: ROS, reactive oxygen species; EV, extracellular vesicle; MMT, mesothelial-to-mesenchymal transition.

The proposed multicellular communication model is summarized in Figure 3.

**Figure 4:**
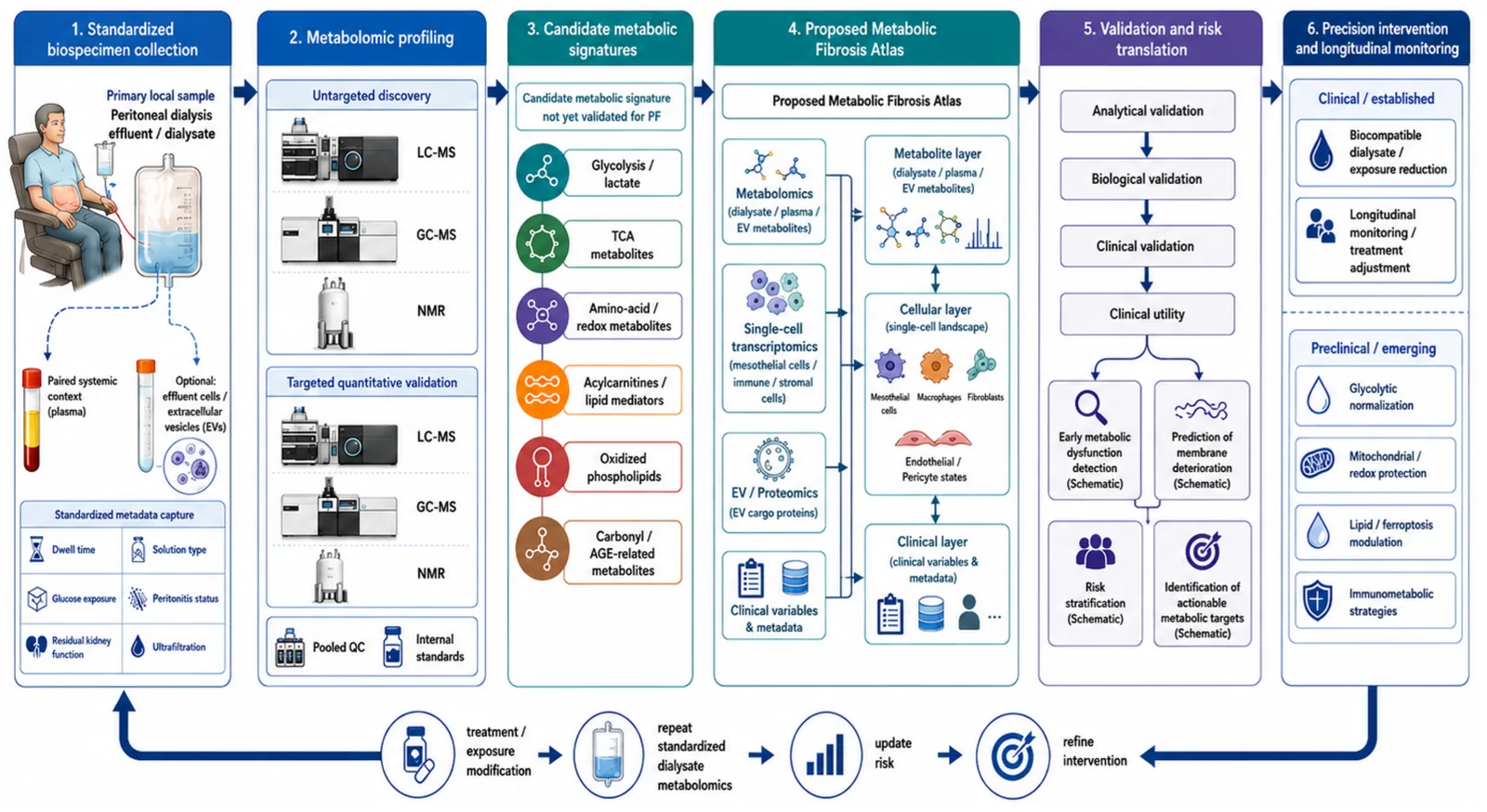
Proposed translational workflow from dialysate metabolomics to precision management. Serial effluent collection, orthogonal analytical platforms, multi-omics integration, risk prediction, and response monitoring form a conceptual closed-loop framework. The prediction curves and treatment-response trajectories are schematic and do not represent empirical study results.

Its proposed clinical translation into longitudinal dialysate profiling is shown in Figure 4.

**Figure 5:**
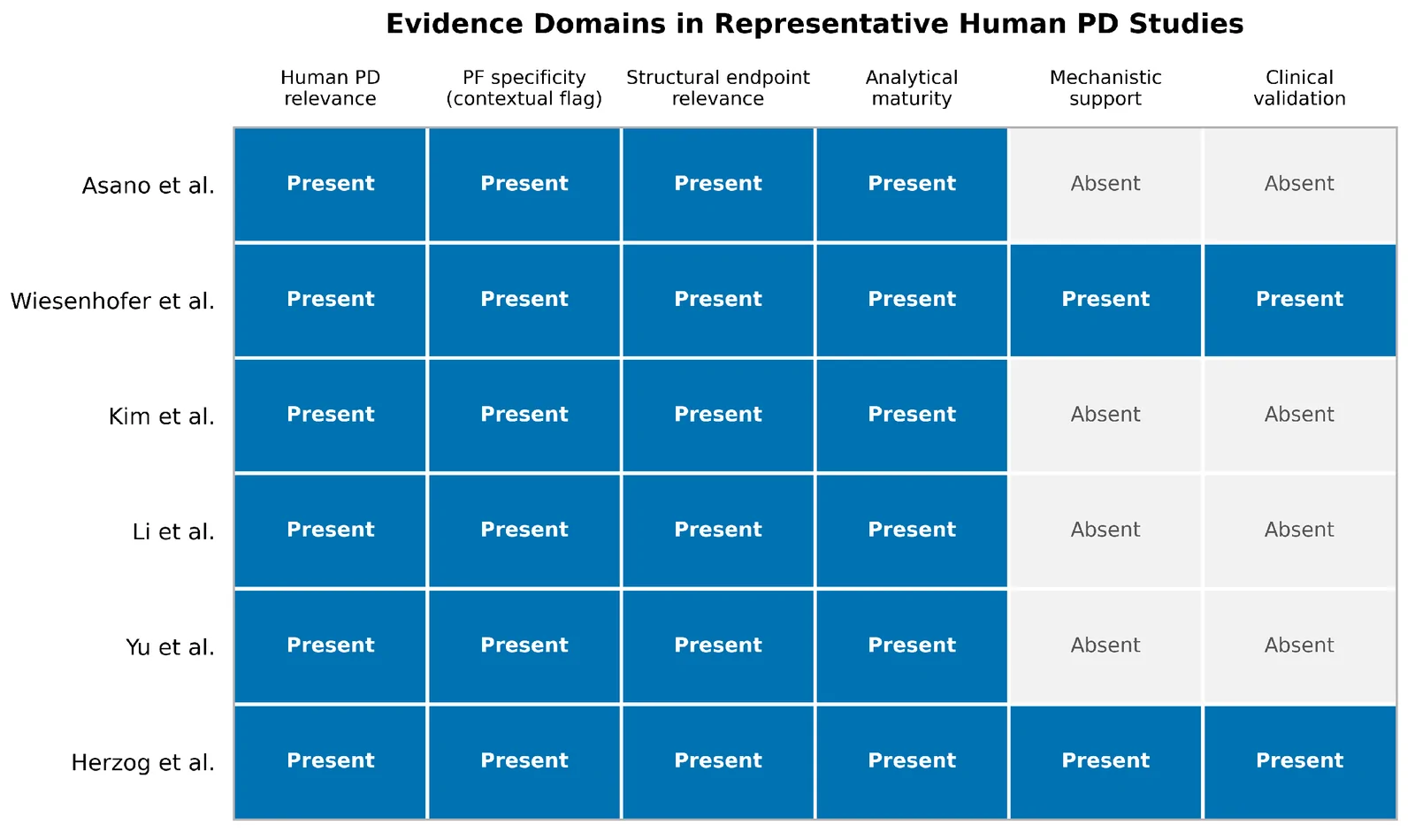
Categorical evidence-domain matrix for the representative human PD studies in Table 1. The studies named in the figure are reported by Asano et al. [3], Wiesenhofer et al. [57], Kim et al. [89], Li et al. [29], Yu et al. [90], and Herzog et al. [58]. The six domains are human PD relevance, PF specificity, structural endpoint relevance, analytical maturity, mechanistic support, and clinical validation. Filled cells indicate evidence present in the published design or outcome; open cells indicate evidence absent or limited. Domains are displayed separately and are not assigned numerical weights, summed, ranked, or interpreted as effect sizes or a validated risk-of-bias instrument. The PF-specificity and structural-endpoint-relevance flags are contextual study-design descriptors and do not imply a PF-specific marker or a Tier I structural PF endpoint; none of these representative studies used histology, validated structural imaging, or clinically adjudicated PF/EPS, and no candidate is called PF-specific in this review.

Figure 5 displays six prespecified human-evidence domains without summing heterogeneous dimensions into a total score. The domains are contextual descriptors of the published study design and endpoint; they are not a quality score, an effect size, or a ranking.

## 8. Future Perspectives and Research Agenda

### 8.1. A Staged Validation Pipeline

The field should move from descriptive metabolite lists to clinically anchored, mechanism-aware panels. Stage 1 is analytical validation: stable-isotope internal standards, recovery controls, batch correction, and inter-laboratory reproducibility. Stage 2 is biological validation: concordance among dialysate, plasma, effluent cells, EVs, and tissue; temporal ordering; and perturbation of the nominated pathway. Stage 3 is clinical validation: prospective cohorts with standardized PET, ultrafiltration, peritonitis history, residual kidney function, and adjudicated PF or EPS outcomes. To establish whether a reversible metabolic phase exists, metabolic changes must precede deterioration in membrane function, reproduce within individuals, associate with structural or clinically adjudicated PF outcomes, and show reversibility after exposure modification or targeted intervention. Stage 4 is utility: demonstration that a metabolite-guided intervention changes membrane preservation or patient-centered outcomes beyond current clinical monitoring.

### 8.2. Longitudinal Sampling and Risk Stratification

A single effluent sample cannot distinguish a stable metabolic phenotype from an acute response to peritonitis, antibiotics, or recent solution change. Repeated sampling at baseline, after a defined dwell, after peritonitis recovery, and at clinically meaningful transport milestones is more informative. Models should include dialysis vintage, glucose exposure, icodextrin use, diabetes, nutrition, inflammation, and residual kidney function. A closed-loop approach would repeat dialysate metabolomics after an intervention and update risk estimates rather than treat the first signature as a permanent patient label. Longitudinal transformer-based models in other aging cohorts illustrate how repeated symptom and clinical measurements can support dynamic risk prediction. This methodological analogy does not establish PD performance; any PD application would require adequately powered prospective cohorts, prespecified feature handling and leakage control, and strict external validation before it could be used to predict membrane dysfunction or PF risk [108].

### 8.3. Causal Inference and Reporting Standards

Metabolomics studies should pre-specify the primary outcome and distinguish discovery from confirmation. Feature selection must occur within the training data; repeated samples from the same patient must not be split across training and test sets; and calibration, decision-curve analysis, and external validation should accompany area under the receiver-operating-characteristic curve. Reports should provide full metadata, quality-control samples, missingness, normalization, multiple-testing correction, and deposited raw or processed data when permitted. For mechanistic papers, metabolite concentration should be complemented by flux, enzyme activity, or rescue experiments. These practices are essential because a statistically strong signature can still be biologically nonspecific or clinically unusable.

### 8.4. Evidence Synthesis and Knowledge Gaps

Table 5 separates human PD observations and support from mechanisms supported mainly by experimental PD models and from the questions that must be resolved before structural PF claims or clinical implementation.

### 8.5. Limitations of the Current Evidence

Several limitations temper the proposed framework. First, direct human evidence linking a metabolic change to histological PF is sparse; many studies use transport phenotype, effluent markers, or animal models as surrogates. Second, dialysis solutions, dwell times, and patient populations differ substantially, complicating cross-study comparisons. Third, ferroptosis, immunometabolism, and EV biology are rapidly evolving fields, and some recent studies are available only as early online publications. Fourth, pathways may be context-dependent: for example, lactate can indicate pathological glycolysis but can also serve as a fuel or signaling metabolite in other settings. Finally, natural products and multi-target agents may improve fibrosis without proving that metabolism was the initiating lesion. These uncertainties argue for triangulation and for explicit separation of evidence tiers rather than for a single unifying biomarker claim.

## 9. Conclusions

Peritoneal dialysis-associated peritoneal fibrosis is best viewed as a multifactorial fibrosis process with an important and increasingly recognized metabolic dimension. High glucose and GDPs, oxidative and carbonyl stress, mitochondrial dysfunction, lipid-peroxidation susceptibility, amino-acid remodeling, and immune-cell metabolic plasticity are interconnected rather than isolated abnormalities. Direct PD studies support hyperglycolysis, effluent metabolomics, mitochondrial and redox injury, and emerging ferroptosis mechanisms; complementary kidney-fibrosis studies provide testable mechanisms but should not be mistaken for direct clinical proof. The most actionable research direction is a longitudinal dialysate-metabolomics program integrated with cellular and clinically adjudicated structural outcomes. Such a program could test whether a clinically detectable metabolic phase precedes fixed membrane fibrosis and whether metabolite-guided selection of biocompatible fluids, metabolic protection, or immunometabolic therapy improves membrane preservation.  

## Figures and Tables

**Table 5:** Current evidence boundaries and priority knowledge gaps in the metabolism-centered framework. This synthesis table summarizes evidence domains and does not replace the study-level numerical extraction in Table 1, Table 2, Table 3 and Table 4.

Domain	Human PD Observations/Support	Supported Primarily by Experimental PD Models	Major Unresolved Questions
Effluent metabolic profiling	Quantitative profiling is feasible, with reported associations involving PET category, peritonitis outcome, and pharmacodynamic response [57,89,90].	Experimental tissue and cell studies connect selected metabolites to pathway activity, but they do not validate a human structural-PF signature.	Can transport, dilution, systemic concentration, and acute inflammation be separated from local production? Will a locked panel replicate against histology, validated structural imaging, or adjudicated PF/EPS?
Glycolytic remodeling	Effluent glucose/lactate and small human-tissue series support association with membrane transport or dysfunction, not causal human PF [16,89].	Mesothelial hyperglycolysis and endothelial PFKFB3 perturbation alter structural and functional peritoneal endpoints [6,16].	Does altered flux precede structural disease in humans, and can it be modulated without impairing mesothelial repair or systemic glucose homeostasis?
Mitochondrial, redox, and fatty-acid pathways	Human effluent studies show pharmacodynamic redox changes and CPT1A-associated cell states, but direct tissue flux data are absent [45,57].	Mitochondrial protection, autophagy modulation, and bidirectional CPT1A perturbation improve experimental endpoints [39,42,45].	Which cell type and compartment drives the signal, and which intervention restores function rather than only lowering oxidative-stress markers?
Ferroptosis	No human study has validated ferroptosis against structural PF; available human observations are marker-level or exposure-context evidence [52,55].	Induced-ferroptosis and PD/PF models provide iron, lipid-peroxidation, pathway-perturbation, and rescue evidence of varying completeness [51,53,54].	Can selective rescue, oxidized-phospholipid profiling, iron dependence, and tissue localization be demonstrated together in non-induced models and then confirmed in human samples?
Immunometabolism and EV signaling	Human studies support inflammatory/peritonitis prediction and the feasibility of effluent EV or multi-omic profiling, not structural PF causality [58,82].	Macrophage-specific perturbation and EV/LDHA interventions reduce experimental remodeling [65,91].	Which cell source, cargo, metabolic pathway, dose, and sampling normalization are reproducible, and do they add value beyond standard clinical monitoring?

## Data Availability

No new data were created or analyzed in this study. This narrative review involved analysis of published literature and generated no new data. If additional information or assistance is required, please contact the corresponding author.

## Abbreviations

The following abbreviations are used in this manuscript:

AGE	advanced glycation end product
CA-125	cancer antigen 125
CKD	chronic kidney disease
DAMP	damage-associated molecular pattern
ECM	extracellular matrix
EPS	encapsulating peritoneal sclerosis
EV	extracellular vesicle
FAO	fatty-acid oxidation
GDP	glucose degradation product
GPX4	glutathione peroxidase 4
HIF-1α	hypoxia-inducible factor-1α
LC–MS	liquid chromatography–mass spectrometry
GC–MS	gas chromatography–mass spectrometry
MMT	mesothelial-to-mesenchymal transition
mtROS	mitochondrial reactive oxygen species
ECAR	extracellular acidification rate
GSH/GSSG	reduced/oxidized glutathione ratio
NLRP3	NOD-like receptor family pyrin domain-containing 3
NF-κB	nuclear factor-κB
NMR	nuclear magnetic resonance
PD	peritoneal dialysis
PET	peritoneal equilibration test
PF	peritoneal fibrosis
ROS	reactive oxygen species
SLC7A11	solute carrier family 7 member 11
STAT3	signal transducer and activator of transcription 3
CPT1A	carnitine palmitoyltransferase 1A
TCA	tricarboxylic acid
TGF-β	transforming growth factor-β

## Appendix A. Database Search Strings

The final searches were completed on 26 August 2026 from database inception, without a language restriction. Table A1 reports the compact database-adapted strings; Google Scholar was used for citation chasing and terminology expansion. Duplicate records were matched by DOI, PMID, title, and first author.

**Table A1:** Database-adapted search strings used for the narrative review. An asterisk (*) denotes a truncation wildcard; for example, metabol* retrieves terms beginning with “metabol” such as metabolite, metabolism, and metabolomics.

Database	Stream A: Metabolic Mechanisms with PF/Membrane Outcomes	Stream B: Human PD Metabolite and Multi-Omic Signals Without a Required PF Term
PubMed	(“Peritoneal Dialysis”[Mesh] OR peritoneal dialysis[tiab]) AND (peritoneal fibrosis[tiab] OR membrane dysfunction[tiab] OR ultrafiltration failure[tiab] OR encapsulating peritoneal sclerosis[tiab]) AND (metabol*[tiab] OR lipidomics[tiab] OR glycolysis[tiab] OR lactate[tiab] OR mitochondri*[tiab] OR fatty acid oxidation[tiab] OR ferroptosis[tiab] OR immunometabolism[tiab])	(“Peritoneal Dialysis”[Mesh] OR peritoneal dialysis[tiab] OR dialysate[tiab] OR effluent[tiab]) AND (metabolomics[tiab] OR lipidomics[tiab] OR metabolite*[tiab] OR proteomics[tiab] OR extracellular vesicle*[tiab])
Embase	(‘peritoneal dialysis’/exp OR ‘peritoneal dialysis’:ti,ab) AND (‘peritoneal fibrosis’:ti,ab OR ‘membrane dysfunction’:ti,ab OR ‘ultrafiltration failure’:ti,ab OR ‘encapsulating peritoneal sclerosis’:ti,ab) AND (metabol*:ti,ab OR lipidomics:ti,ab OR glycolysis:ti,ab OR lactate:ti,ab OR mitochondri*:ti,ab OR ‘fatty acid oxidation’:ti,ab OR ferroptosis:ti,ab OR immunometabolism:ti,ab)	(‘peritoneal dialysis’/exp OR ‘peritoneal dialysis’:ti,ab OR dialysate:ti,ab OR effluent:ti,ab) AND (metabolomics:ti,ab OR lipidomics:ti,ab OR metabolite*:ti,ab OR proteomics:ti,ab OR ‘extracellular vesicle*’:ti,ab)
Web of Science Core Collection	TS=((“peritoneal dialysis”) AND (“peritoneal fibrosis” OR “membrane dysfunction” OR “ultrafiltration failure” OR “encapsulating peritoneal sclerosis”) AND (metabol* OR lipidomics OR glycolysis OR lactate OR mitochondri* OR “fatty acid oxidation” OR ferroptosis OR immunometabolism))	TS=((“peritoneal dialysis” OR dialysate OR effluent) AND (metabolomics OR lipidomics OR metabolite* OR proteomics OR “extracellular vesicle*”))
Google Scholar	“peritoneal dialysis” (“peritoneal fibrosis” OR “membrane dysfunction” OR “ultrafiltration failure”) (metabolomics OR lipidomics OR “metabolic remodeling” OR glycolysis OR mitochondria OR ferroptosis OR immunometabolism)	“peritoneal dialysis” (dialysate OR effluent) (metabolomics OR lipidomics OR metabolites OR proteomics OR “extracellular vesicles”); both streams were supplemented by forward and backward citation chasing.

## Appendix B. Evidence-Domain Coding

Figure 5 displays six prespecified evidence domains for the representative human studies in Table 1: human PD relevance, PF specificity, structural endpoint relevance, analytical maturity, mechanistic support, and clinical validation. Domains were coded categorically as evidence present or absent/limited from the published design and outcomes; they were not assigned numerical weights or summed. PF specificity and structural endpoint relevance are contextual flags and do not substitute for a Tier I structural PF endpoint. This matrix is an evidence map, not a validated risk-of-bias instrument. Table A2 provides the operational definition of each domain.

**Table A2:** Definitions used for the categorical evidence-domain matrix in Figure 5.

Evidence Domain	Present When the Primary Study Reported
Human PD relevance	Direct study of human PD participants or PD-derived effluent, cells, tissue, or clinical samples; systemic comparator samples alone did not meet this domain.
PF specificity (contextual flag)	The study question or measured biology was explicitly linked to peritoneal membrane injury, PF-related remodeling, or a PF-relevant pathway. For observational effluent studies this is a contextual design flag only; it does not establish discrimination from transport changes, inflammation, peritonitis, or other membrane injury. A PF-specific marker additionally requires a Tier I structural or clinically adjudicated PF/EPS outcome, prespecified adjustment for relevant confounders, and independent replication, as defined in Section 2.3 and Section 6.1.
Structural endpoint relevance	The study reported a membrane structure or clinically relevant membrane phenotype (for example, PET transport, ultrafiltration, membrane thickening, or a related structural correlate). A surrogate phenotype was coded as relevant but not as a Tier I structural PF endpoint.
Analytical maturity	The study used a reportable quantitative assay or profiling platform with analyte-level output and described essential analytical procedures, such as calibration, quality control, or reproducibility checks, when applicable.
Mechanistic support	The design included pathway perturbation, a controlled exposure, target engagement, or a prespecified biological mediation analysis beyond descriptive association.
Clinical validation	The human signal was evaluated prospectively, in a controlled/interventional clinical design, or in an independent or multicenter validation cohort. A single cross-sectional association was coded absent or limited.
